# A novel clip treatment for gastrointestinal bleeding in hereditary hemorrhagic telangiectasia

**DOI:** 10.1055/a-2878-1094

**Published:** 2026-06-19

**Authors:** Robbie Chan, Hirannya Karunadasa, Ronan O'Connor, Mark Lai, Paul Urquhart

**Affiliations:** 1 Department of Gastroenterology 1890 Eastern HealthMelbourneVictoriaAustralia

Hereditary hemorrhagic telangiectasia (HHT) is an autosomal dominant disorder characterised by the presence of abnormal blood vessel formations which can cause gastrointestinal bleeding in 20% of patients. 1​ Bleeding arteriovenous malformations (AVMs) can be managed with endoscopic and pharmacological therapies. We present the first case report of over-the-scope (OTS) clip use to treat refractory gastrointestinal AVMs in a patient with HHT.

A 42-year-old man presented with progressive anemia on a background of HHT with transfusion requirements of up to eight units of packed red blood cells weekly. The patient experienced refractory gastrointestinal bleeding despite extensive systemic therapy including tranexamic acid, thalidomide, pomalidomide, and bevacizumab. He had received repeated endoscopic intervention for bleeding gastric and duodenal AVMs with argon plasma coagulation (APC) and through-the-scope (TTS) endoclips through push enteroscopy, antegrade double balloon enteroscopy, and surgically assisted enteroscopy. OTS clips were considered as an alternative treatment.

**Fig. 1:**
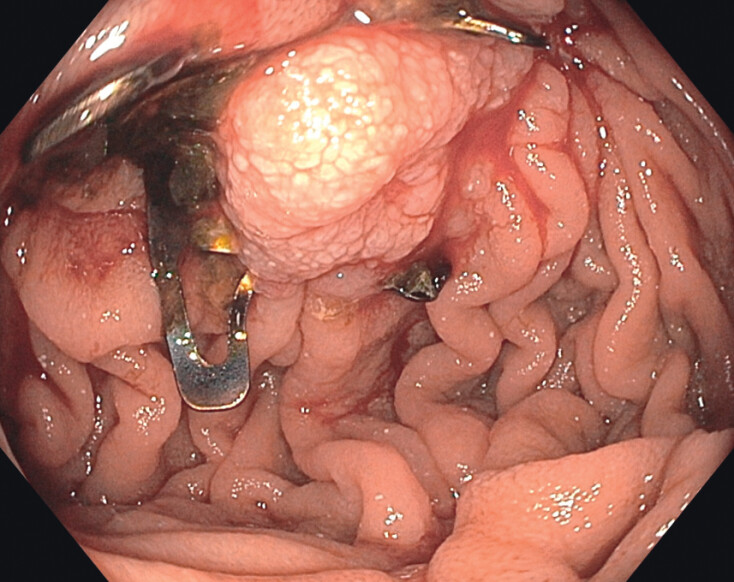
OTS clips applied to the duodenal arteriovenous malformation. OTS, over-the-scope.

Elective endoscopy revealed a large actively bleeding AVM in the D3 segment of the duodenum. An OTS clip was applied ( Video 1 and Fig. 1 ). The area was further treated with hemostatic powder, achieving hemostasis.

**Video 1** OTS clips deployed to duodenal and gastric arteriovenous malformations with hemostasis achieved following combination therapy.


**Fig 2:**
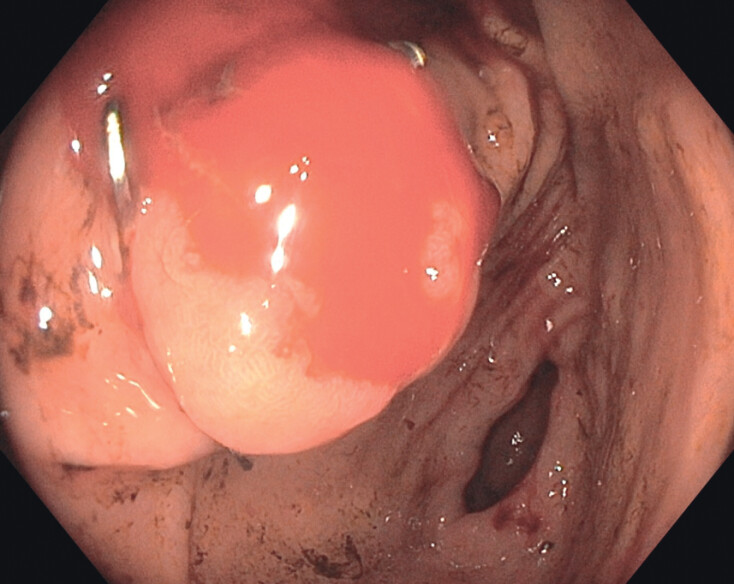
A further OTS clip applied to the gastric arteriovenous malformation. OTS, over-thescope.

On repeat endoscopy 4 weeks later, examination of the OTS clip site showed no further active bleeding. Two further OTS clips were applied to duodenal and gastric ulcers ( Fig. 2 ), in combination with ablation and TTS endoclips. Following this treatment, the patient’s transfusion requirements improved to two to three units of packed red blood cells weekly.

In patients with HHT with recurrent GI bleeding, endoscopy is recommended to identify culprit lesions and administer local therapy, usually with APC. 2​ Endoscopic treatment of gastrointestinal AVMs is effective in the long term, 3​ allowing patients to avoid potential adverse effects of systemic therapies.

The use of OTS clips on selected lesions may be an alternative means of treating refractory gastrointestinal bleeding which has not responded to standard endoscopic intervention in patients with HHT.

Endoscopy_UCTN_Code_TTT_1AO_2AD
